# Characterising cancer-associated fibroblast heterogeneity in non-small cell lung cancer: a systematic review and meta-analysis

**DOI:** 10.1038/s41598-021-81796-2

**Published:** 2021-02-12

**Authors:** Andrew F. Irvine, Sara Waise, Edward W. Green, Beth Stuart, Gareth J. Thomas

**Affiliations:** 1 School of Cancer Sciences, Faculty of Medicine, University of Southampton, Southampton, UK; 2 The German Cancer Research Centre (DKFZ), Heidelberg, Germany; 3 Present Address: Department of Pathology and Data Analytics, University of Leeds, Leeds, UK; 4 Primary Care and Population Sciences, Faculty of Medicine, University of Southampton, Southampton, UK

**Keywords:** Non-small-cell lung cancer, Cancer microenvironment, Prognostic markers

## Abstract

Cancer-associated fibroblasts (CAFs) are a key component of the tumour microenvironment with evidence suggesting they represent a heterogeneous population. This study summarises the prognostic role of all proteins characterised in CAFs with immunohistochemistry in non-small cell lung cancer thus far. The functions of these proteins in cellular processes crucial to CAFs are also analysed. Five databases were searched to extract survival outcomes from published studies and statistical techniques, including a novel method, used to capture missing values from the literature. A total of 26 proteins were identified, 21 of which were combined into 7 common cellular processes key to CAFs. Quality assessments for sensitivity analyses were carried out for each study using the REMARK criteria whilst publication bias was assessed using funnel plots. Random effects models consistently identified the expression of podoplanin (Overall Survival (OS)/Disease-specific Survival (DSS), univariate analysis HR 2.25, 95% CIs 1.80–2.82) and α-SMA (OS/DSS, univariate analysis HR 2.11, 95% CIs 1.18–3.77) in CAFs as highly prognostic regardless of outcome measure or analysis method. Moreover, proteins involved in maintaining and generating the CAF phenotype (α-SMA, TGF-β and p-Smad2) proved highly significant after sensitivity analysis (HR 2.74, 95% CIs 1.74–4.33) supporting attempts at targeting this pathway for therapeutic benefit.

## Introduction

Non-small cell lung cancer (NSCLC) remains the leading cause of cancer death worldwide^1–3^. Despite more recent therapeutic advances, outcomes remain poor, with a 10-year survival rate of only 5%^4^. NSCLC shows a relatively low degree of tumour cell purity compared to other tumours, with high infiltration by immune and stromal cell populations^5^.

Fibroblasts are the most common stromal cell type in a range of solid tumours^6–9^, where they are referred to as cancer-associated fibroblasts (CAFs). CAFs are most commonly described as having an α-SMA-positive, “myofibroblastic” phenotype, analogous to that observed in wound healing^10^. These cells are associated with a number of the hallmarks of malignancy, including promotion of tumour invasion and metastasis, angiogenesis and immune evasion^11–18^. Unsurprisingly, this population correlates with poor prognosis in a range of malignancies^9, 19–21^. However, there is increasing evidence that CAFs are in fact a heterogeneous cell type, with a range of distinct phenotypes and functions^22–24^. For example, an inflammatory CAF sub-group has been described in a number of different tumours including pancreatic cancer^25^. Nevertheless, the relative contribution of specific populations is likely to vary by tumour type and has yet to be defined fully and for some cancer types, including NSCLC, the impact of CAFs on patient outcomes is less clear.

α-SMA is the most commonly used CAF marker^26^, but is also expressed by smooth muscle cells^27^ and pericytes^28^ and no single marker has been shown to reliably identify the entire CAF population. Indeed, FAP, another commonly-used CAF marker has been shown to identify both inflammatory and myofibroblastic CAFs in pancreatic ductal adenocarcinoma^17^ and breast cancer^18^. Other frequently used CAF markers include podoplanin and fibroblast-specific protein-1 (FSP-1)^9, 29^ with CAFs expressing the latter known to have immunomodulatory functions^30–32^. However, for others, the downstream functional pathways are yet to be characterised.

CAFs are an attractive therapeutic target, but despite promising data from pre-clinical models, the results of clinical trials targeting CAFs have been mixed^33, 34^. Characterisation of CAF phenotypes and their impact on outcomes has gained increasing interest in recent years, and there are now multiple studies profiling CAF heterogeneity at single-cell resolution^22–24^. To date, there have been many individual reports describing the prognostic effect of single CAF markers in NSCLC. The impact of CAFs seems to vary by marker and, in some cases, is contradictory (e.g. FAP^35, 36^). This may be explained, at least in part, by the known heterogeneity within this population, where common markers can be expressed by functionally distinct subgroups.

Although meta-analyses examining the relationship between protein marker expression and outcomes in NSCLC have been performed previously^37, 38^, these studies only focused on a small number of pre-determined markers and did not use methods to extract hazard ratios from studies which failed to quote them, leading to possible publication bias. Moreover, the number of studies published in the intervening period has increased significantly reflecting the increased interest in CAFs. Here, we perform a systematic review and meta-analysis of the literature assessing the prognostic effect of all CAF markers in NSCLC characterised thus far, as well as the cellular processes they are involved in. In addition, we also use several statistical methods, including a newly-published method^39^ which improves on the accuracy of extracted HRs when not quoted in the original studies. Assessing the prognostic significance of these markers is important in characterising the heterogeneity now widely accepted in CAFs whilst determining the most prognostic cellular pathways might help inform stromal targeting strategies in NSCLC.

## Results

### Study characteristics

**Figure 1:**
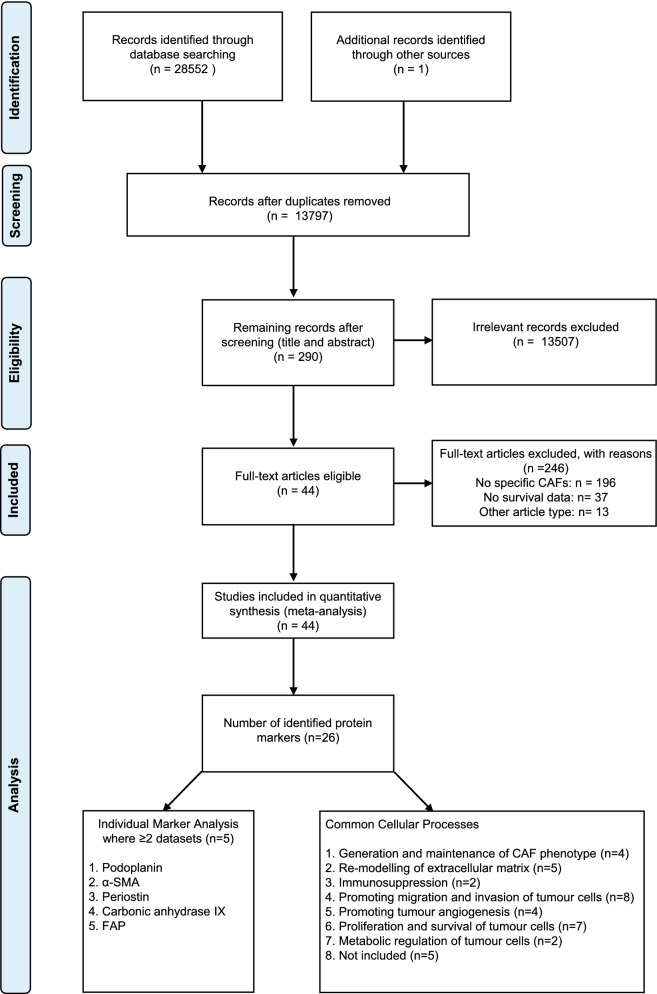
Flow chart describing steps carried out in selecting articles.

Of the 13,797 articles identified, 290 were eligible after screening titles and abstracts. Of these, 44 were included based on the full-text article, representing a total population of 7582 patients (Fig. 1). Cross-checking of previously published reviews on the roles of fibroblasts in lung cancer yielded one additional study that was not detected within the search strategy^40^. Of the 246 studies excluded, 196 described protein expression within the wider microenvironment, rather than specifically by fibroblasts which was the most common reason for study exclusion. A further 37 studies did not include survival statistics or a Kaplan–Meier (KM) plot from which these could be calculated. Twenty-six protein markers were identified from the 44 included articles. Five of these (podoplanin, carbonic anhydrase IX (CAIX), α-SMA, periostin and FAP) appeared in at least 2 separate cohorts, rendering them suitable for meta-analysis. Of all included markers, 21 (81%) were identified as a component of at least one common cellular process that defines, or is a hallmark, of cancer-associated fibroblasts^41^.

The main characteristics of the included studies are shown in Supplementary Table S1. The earliest included study was published in 1998. However, the majority (30/44, 68%) were published within the last 5 years, likely reflecting the increased interest in CAFs. The median cohort size was 129 (range 52–729). Many studies reported cohorts focusing on lung adenocarcinoma (19/44, 43%) or a combination of adenocarcinoma and squamous cell carcinoma (17/44, 39%). In terms of treatment, 19 studies (43%) failed to report information on neo- or adjuvant therapy whilst 13 studies (30%) excluded patients who had received neo-adjuvant therapy. Almost half of studies (21/44, 48%) reported overall survival (OS) as the only survival outcome. The majority of studies reported a mix of univariate and multivariate hazard ratios (HR) (24/44, 55%) with 21/44 studies (48%) reporting a KM plot but no associated HR. To extract these missing HRs, we used a set of statistical techniques depending on the available information, including the Nlopt method^39^, a novel algorithm based on non-linear optimisation (see “Methods” section for more details). In these cases, the Nlopt method was used most frequently (14/21, 67%), with the Parmar^42^ (3/21, 14%) and Guyot^43^ method (4/21, 19%) also required in several instances. In total, the 44 included studies yielded 96 survival outcome measures.

### Quality assessment

Assessment of study quality was determined by calculating a score based on the REMARK criteria^44, 45^ (summarised in Supplementary Figure S1; raw data given in Supplementary Table S2). The mean score was 14 (range 9.5–17) with most studies scoring moderately well against all domains of the REMARK criteria. The exception was the “data” domain in which all but one study^46^ registered low- to medium-quality scores. The data domain describes the flow of patients through the original study, as well as the relationship of the tumour marker to standard prognostic variables. In total, 3 studies had overall REMARK scores ≤ 50%, all of which were included in subsequent sensitivity analyses. Although the REMARK criteria were first published in 2005, in the studies included in this systematic review, there has not been a significant increase in scores over this time (R^2^ = − 0.017, *P* = 0.603; Supplementary Figure S2).

### Individual marker results

**Figure 2:**
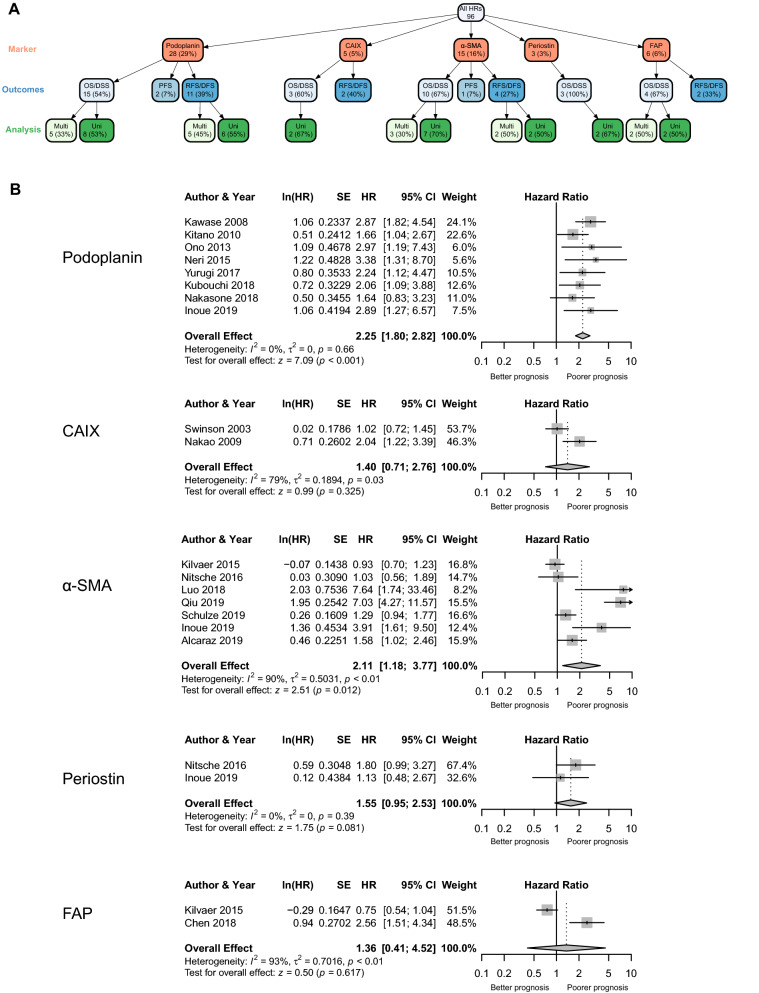
Analysis of individual markers. (**A**) Tree network showing number of studies for each marker per outcome group and analysis method. Figure generated using the *vtree* package in R (version 3.5.2). (**B**) Random-effect forest plots of individual markers from the OS/DSS outcome group and univariate analysis method.

Podoplanin, α-SMA, FAP, periostin and CAIX all had at least two HRs calculated using similar outcome measures and assessed using either univariate or multivariate analysis deeming them eligible for meta-analysis. Calculating these separately was recommended in guidance published on carrying out meta-analyses on prognostic factors^47^. This approach resulted in twelve separate outcome measures of pooled HRs analysed using a random effects model as represented in the network tree (Fig. 2A). Example forest plots for univariate analysis of the OS/DSS outcome group for each marker are shown in Fig. 2B; full results are summarised in Table 1.

**Table 1:** Summary of results from the random effects models for individual markers.

Marker	Outcomes	Analysis	Studies	Random effects model		Heterogeneity		
				Overall effect (95% CIs)	*P* value	I^2^ (%)	τ^2^	*P* value
Podoplanin	OS/DSS	Univariate	8	2.25 (1.80–2.82)	< 0.001	0	0.00	0.66
		Multivariate	5	1.67 (1.03–2.73)	0.040	67	0.19	0.02
	RFS/DFS	Univariate	6	2.73 (2.11–3.54)	< 0.001	0	0.00	0.59
		Multivariate	5	2.19 (1.54–3.12)	< 0.001	0	0.00	0.41
CAIX	OS/DSS	Univariate	2	1.40 (0.71–2.76)	0.320	79	0.19	0.03
α-SMA	OS/DSS	Univariate	7	2.11 (1.18–3.77)	0.012	90	0.50	< 0.01
		Multivariate	3	3.21 (1.45–7.10)	0.004	71	0.31	0.03
	RFS/DFS	Univariate	2	8.12 (5.23–12.62)	< 0.001	0	0.00	0.93
		Multivariate	2	5.38 (3.34–8.67)	< 0.001	0	0.00	0.69
Periostin	OS/DSS	Univariate	2	1.55 (0.95–2.53)	0.081	0	0.00	0.39
FAP	OS/DSS	Univariate	2	1.36 (0.41–4.52)	0.620	93	0.70	< 0.01
		Multivariate	2	2.25 (1.39–3.63)	0.001	0	0.00	0.68

Podoplanin and α-SMA were the most frequently reported of the five markers and were consistently associated with statistically significant poorer survival outcomes, regardless of outcome measure or analysis method (Table 1). However, significant test heterogeneity was found in a subset of these measures. As expected, HRs for RFS/DFS were always higher than OS/DSS although no other trends emerged when comparing survival outcomes from different groups. In contrast, CAIX (HR 1.40, 95% CI 0.71–2.76) and periostin (HR 1.55, 95% CI 0.95–2.53) did not show a significant correlation with survival. FAP expression was only associated with a statistically significant poor prognostic impact in the multivariate analysis from the OS/DSS outcome group (HR 2.25, 95% CI 1.39–3.63).

**Figure 3:**
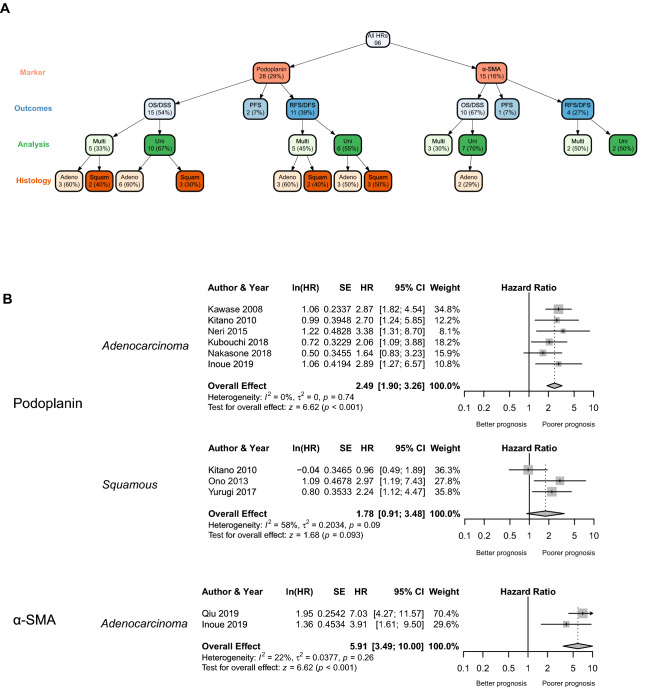
Sub-group analysis of individual markers based on histological subtype. (**A**) Tree network showing number of studies for each marker per outcome group, analysis method and histological subtype. Figure generated using the *vtree* package in R (version 3.5.2). (**B**) Random-effect forest plots of individual markers from the OS/DSS outcome group and univariate analysis method with histological subtype indicated.

In all eight podoplanin and one α-SMA random effects models, there were a sufficient number of studies to carry out sub-group analysis based on histological subtype (Fig. 3A,B, Table 2). In the case of podoplanin, all were significantly associated with a poorer survival outcome, with the exception of multivariate analysis of the OS/DSS outcome group in adenocarcinoma and univariate analysis of the OS/DSS outcome group in squamous cell carcinoma. The univariate analysis of α-SMA in a cohort of patients with only adenocarcinoma was statistically significant (HR 5.91, 95% CI 3.49–10.00).

**Table 2:** Subgroup analysis: summary of results from the random effects models for individual markers based on histology sub-type.

Marker	Outcomes	Analysis^a^	Histology^b^	Studies	Random Effects Model		Heterogeneity		
					Overall effect (95% CIs)	*P* value	I^2^ (%)	τ^2^	*P* value
Podoplanin	OS/DSS	U	A	6	2.49 (1.90–3.26)	< 0.001	0	0.00	0.74
			S	3	1.78 (0.91–3.48)	0.093	58	0.20	0.093
		M	A	3	1.28 (0.67–2.45)	0.460	68	0.20	0.05
			S	2	2.50 (1.58–3.95)	< 0.001	0	0.00	0.59
	RFS/DFS	U	A	3	3.12 (2.17–4.49)	< 0.001	10	0.01	0.33
			S	3	2.30 (1.55–3.42)	< 0.001	0	0.00	0.89
		M	A	3	2.59 (1.19–5.65)	0.020	46	0.22	0.16
			S	2	2.04 (1.30–3.18)	0.002	0	0.00	0.94
α-SMA	OS/DSS	U	A	2	5.91 (3.49–10.00)	< 0.001	22	0.04	0.26

^a^*U* univariate, *M* multivariate.

^b^*A* adenocarcinoma, *S* squamous.

After sensitivity analysis excluding studies with low REMARK scores, two scores for podoplanin (OS/DSS > Univariate > All histology and OS/DSS > Univariate > adenocarcinoma only) remained unchanged (Supplementary Table S3).

### CAF markers in cellular processes

**Figure 4:**
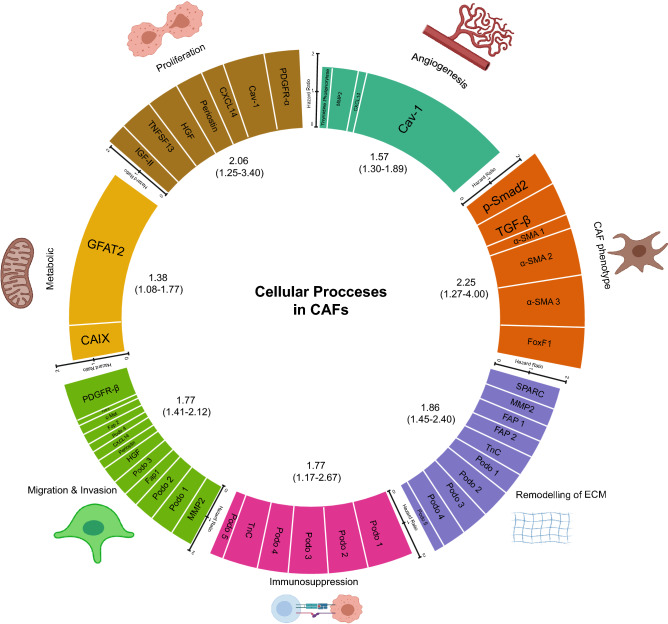
Ferris wheel plot summarising random-effect model HRs for each cellular process in CAFs. The height of each bar represents the HR for each process with the width of each bar indicating the % weight that each marker contributed to the random-effects model. The random-effect model HRs and 95% CIs are stated below each cellular process. Figure generated using Adobe Illustrator, 2020 (version 24.2). Icons representing each cellular process are from BioRender.com.

Our next aim was to assess the prognostic significance of the cellular processes known to be crucial in the function of CAFs. CAFs are known to have a variety of functions which influence cancer progression and which have been summarised in a number of recent reviews^26, 41, 48^. We established a consensus of functions from these reviews, creating a table of what are currently considered the most important functions or hallmarks of CAFs (Supplementary Table S4). Next, to determine the function of each of the identified markers, a separate literature search was performed focusing on studies which had identified a functional role of the marker specifically in CAFs. In several cases, this came from the functional studies published in the original paper, for example, c-Met^49^, GFAT2^50^ and IGF-II^51^. Such studies generally included co-culture experiments in vitro or more complex mouse models in vivo or a combination of the two (Supplementary Table S5). Each of the markers was then assigned to one or more of the functions (Table 3). In two cases, CD34 and irisin have no clear role in CAFs currently so were excluded from this step of the analysis. In addition, three other markers, CD90, HSF-1, CD200 did not have outcome measures in the OS/DSS group so were also excluded from the analysis (see “Methods” section for more detail). In total, this resulted in 21/26 of the markers being attributed to at least one of 7 common processes. Analysis of the pooled HR for each process showed all were in fact associated with poorer survival (Table 4, Fig. 4) although generation and maintenance of the CAF phenotype (HR 2.25, 1.27–4.00 95% CIs) and enhancing the proliferation and survival of tumour cells (HR 2.06, 1.25–3.40 95% CIs) were the only processes with a HR above 2. A significant level of heterogeneity was again detected in 4 of the 7 cellular processes but in the case of the CAF phenotype, this was non-significant after sensitivity analysis excluding poor quality studies (Supplementary Table S3) and the pooled HR in fact increased to 2.74 (1.74–4.33 95% CIs).

**Table 3:** Summary of the CAF functions and the role of each identified marker.

CAF function	Identified markers
1. Generation and maintenance of CAF phenotype	α-SMA, p-Smad2, TGF-β, FoxF1
2. Re-modelling of extracellular matrix	FAP, MMP2, SPARC, Tenascin-C, Podoplanin
3. Immunosuppression	Podoplanin, Tenascin-C
4. Promoting migration and invasion of tumour cells	c-Met, FAP, HGF, MMP2, PDGFR-β, Periostin, Podoplanin, CXCL14
5. Promoting tumour angiogenesis	Caveolin-1, CXCL14, MMP2, Thymidine Phosphorylase
6. Proliferation and survival of tumour cells	Caveolin-1, CXCL14, HGF, IGF-II, PDGFR-α, Periostin, TNFSF13
7. Metabolic regulation of tumour cells	Carbonic anhydrase IX, GFAT2
8. Not included	
No known function in CAFs currently	CD34, Irisin
No outcome measure for OS/DSS	CD90, HSF-1, CD200

**Table 4:** Summary of results from the random effects models for each cellular process. The list of proteins making up each cellular process can be found in Table 3.

Cellular process	Studies	Random effects model		Heterogeneity		
		Overall effect (95% CIs)	*P* value	I^2^ (%)	τ^2^	*P* value
Generation and maintenance of CAF phenotype	6	2.25 (1.27–4.00)	0.006	81	0.36	< 0.01
Re-modelling of extracellular matrix	10	1.86 (1.45–2.40)	< 0.001	41	0.07	0.08
Immunosuppression	6	1.77 (1.17–2.67)	0.007	63	0.16	0.02
Promoting migration and invasion of tumour cells	13	1.77 (1.41–2.12)	< 0.001	46	0.07	0.04
Promoting tumour angiogenesis	4	1.57 (1.30–1.89)	< 0.001	0	0.00	0.50
Enhancing proliferation and survival of tumour cells	7	2.06 (1.25–3.40)	0.005	85	0.34	< 0.01
Metabolic regulation of tumour cells	2	1.38 (1.08–1.77)	0.010	20	0.01	0.26

As with all meta-analyses, small-study effects should be examined to determine the extent of any publication bias. We produced funnel plots and tested asymmetry with linear regression in any meta-analysis with 7 or more studies (Supplementary Figure S3) due to the low power of these tests^52^. In the case of funnel plots for the univariate analysis of α-SMA in the OS/DSS outcome group and the invasion and proliferation meta-analysis (Supplementary Figure S3B, D, E), the plots were clearly asymmetrical but likely due in part to the heterogeneity that was also detected (Tables 1, 4). Only one of the plots was deemed to significantly deviate from a symmetrical distribution (cellular processes: invasion, *P* = 0.02) although this is in part likely due to the heterogeneity mentioned above. However, visual assessment of this funnel plot suggested some values missing where small studies with larger standard errors would be expected suggesting an element of publication bias in this particular random effects model.

## Discussion

CAFs are a key component of the tumour microenvironment. Growing evidence suggests they are a heterogeneous population with respect to function^53^ and expression of both RNA and protein^22, 54^. We therefore performed a meta-analysis of all published protein markers of CAFs in NSCLC in an attempt to better characterise this heterogeneity by determining their prognostic significance. We implemented a search strategy focused on sensitivity, resulting in a number needed to read of 313. In addition, we calculated HRs from studies when these were not directly quoted using the most up-to-date extraction methods. In total, this yielded 26 different protein markers. These included well known markers of CAFs including α-SMA and FAP^26^, but also new potential markers identified from omic-type screens such as CD200^55^ and GFAT2^50^. To ensure that this analysis was fibroblast-specific, we excluded studies which did not explicitly state if the protein marker was expressed by fibroblasts or just more generally within the stroma or tumour microenvironment.

Podoplanin is the best characterised of the identified markers, and in keeping with previous data^37^, was often associated with poor survival in this study. Podoplanin is a 44-kDa glycoprotein that was initially characterised as a platelet-aggregation factor on cancer cells from colorectal tumours^56^. It is also expressed by both lymphatic endothelium^57^ and inflammatory macrophages^58^. Functionally, podoplanin-positive fibroblasts in cancer have been shown to enhance the invasive properties of carcinoma cells^59^, play an important role in re-modelling of the ECM^60–62^, as well as promoting an immunosuppressive microenvironment^63^. Although podoplanin was consistently associated with poor survival in this study, a significant test heterogeneity was detected for the OS/DSS outcome group in multivariate analysis but when sub-grouping based on histological variant was carried out this became non-significant. The same analysis also revealed that squamous but not adenocarcinoma tumours were significantly associated with survival suggesting a possible difference in prognostic effect based on NSCLC histological subtype, explaining the heterogeneity originally identified. However, this trend was not observed in the RFS/DFS outcome measure suggesting further comparisons of podoplanin-positive fibroblasts in squamous and adenocarcinoma tumours are warranted.

α-SMA was also commonly reported and associated with a poorer survival. α-SMA is a member of a highly conserved group of proteins that regulate the cell cytoskeleton^64^. In fibroblasts, this protein is crucial in regulating the contractility of the cell which is itself required to both generate and maintain the CAF phenotype^65^. Although α-SMA was associated with poor survival, it tended to result in pooled HRs with larger variances. For example, in the case of analysing univariate HRs from OS/DSS outcomes, HRs ranged from 0.9 for Kilvaer et al*.*^35^ to over 7 for Qiu et al*.*^66^ whilst contributing similar weights to the random effects model. In comparing these two studies at either extreme, both featured cohorts of patients with stage I-IIIA NSCLC, excluding those treated with neo-adjuvant therapy and scored well on the REMARK criteria. Qiu et al*.*^66^ focused solely on adenocarcinoma cases with Kilvaer et al*.*^35^ considering both squamous cell carcinoma and adenocarcinoma. However, both studies used different immunohistochemistry scoring systems: Kilvaer calculated the dominant staining intensity in positive cells whereas the Qiu study used an index combining both staining intensity and extent. Such a difference in scoring might explain, at least in part, the variation seen in these two studies. Indeed, the use of different scoring criteria in biomarker research and subsequent difficulty in comparing studies is well known^47^, leading for calls to ensure scoring for markers is standardised^67^, and validates the need for a meta-analysis.

Three other markers were also suitable for meta-analysis in this study, CAIX, periostin and FAP. Unlike for α-SMA and podoplanin, fewer studies have been carried out on these markers so there was only one combination of outcome measures and forms of analysis for CAIX and periostin and two for FAP.

CAIX is a member of the carbonic anhydrase family, the expression of which is induced under hypoxic conditions by HIF-1^68^. Whilst hypoxia is clearly an important aspect of tumourigenesis^69^ with the tumour microenvironment (including CAFs) playing an important role in its regulation^70^, CAIX expression in CAFs was not associated with reduced survival in this meta-analysis.

Periostin, an ECM protein produced by fibroblasts^71^, has previously been shown to enhance the proliferation and invasive potential of tumour cells^72^ but was also not associated with poor survival in NSCLC. In the case of FAP, meta-analysis produced conflicting results.

FAP is a type II integral membrane serine protease shown to be involved in ECM re-modelling and tumour cell migration^73^ and has been used as a marker of activated fibroblasts in a number of studies. In this meta-analysis, random effects models showed univariate analysis had no significant effect on prognosis whilst multivariate analysis did prove to be statistically significant. Notably, one of the univariate studies actually showed that increased CAF FAP expression was associated with improved survival in NSCLC^46^. Whilst not statistically significant in a mixed cohort, sub-group analysis of only squamous carcinoma cases was statistically significant, in contrast to findings of Chen et al*.*^74^ In what is emerging as a common theme, these two studies used different antibodies, grading systems and scored either whole slides or tissue micro-arrays; this may account for the discordant results in these studies. Such discrepancies are concerning though as FAP is currently regarded as a CAF target for molecular-based imaging^75^ and therapeutic targeting^76^. Without a clear understanding of its role in CAF biology, such trials might produce inconsistent results.

Some CAF markers were only analysed in a single study or did not share a common outcome group/form of analysis and were therefore excluded from the meta-analysis. These included several interesting studies; Chen et al.^51^ showed high expression of IGF-II in CAFs in a cohort of 80 patients resulted in a HR of 19.15 (95% CIs 6.32–58) for overall survival. In this study, CAFs from primary tumours were shown to promote stemness characteristics of lung cancer-stem cells (expression of Nanog and Oct3/4), an effect which was shown to be partly dependent on the expression of IGF-II. IGF-II in CAFs is known to accelerate tumour growth in cholangiocarcinoma xenograft models^77^ and promotes proliferation of anal squamous cell carcinoma cells^78^. Moreover, expression of IGF-II has been shown to promote differentiation of fibroblasts into myofibroblasts in idiopathic pulmonary fibrosis and scleroderma/systemic sclerosis-associated pulmonary fibrosis^79^. Together, this result suggests further examination of IGF-II expression in CAFs in NSCLC could lead to key biological pathways being elucidated or identification of additional sets of patients with poor survival.

Cav-1, a scaffold protein crucial to caveolae^80^ was also excluded from meta-analysis as the two studies which analysed its prognostic role only calculated either a univariate or multivariate hazard ratio. Along with FAP, Cav-1 was the only other marker that resulted in statistically significant outcome measures with opposite effects on survival. In the study by Shimizu et al*.*^81^, high expression of Cav-1 was associated with a decrease in overall survival (HR 2.78) whilst the study by Onion et al*.*^82^ showed high expression of Cav-1 was associated with improved survival (HR 0.64). The studies used different antibodies and scoring systems whilst the cohort used in the Shimizu study was larger and consisted of only patients with Stage I adenocarcinoma. The Onion study featured a cohort with Stage I–III NSCLC but did not state the histological classification of the tumours included. Given this, it is possible that the differences are due to histological subtype if the cohort in the Onion study was mainly composed of cases of squamous NSCLC. Loss of Cav-1 has previously been shown to correlate with poor survival in other cancers, for example prostate^83^ and breast^84^, in agreement with the Onion study but given the discrepancies identified in this analysis, further studies assessing the prognostic role of Cav-1 in CAFs in NSCLC would be warranted to clarify its prognostic role in lung cancer.

A variety of functions have been attributed to CAFs in recent years, leading to the question of whether all CAFs perform these functions, or whether there exist subsets of CAFs with different functions.

CAFs are increasingly recognised as a heterogeneous cell type. Recent studies have described transcriptomically-distinct CAF phenotypes in NSCLC, which may correspond to discrete functional subsets^22, 23^. Our aim was to therefore determine whether a set of protein markers, grouped together by function, would show prognostic differences. This in turn may suggest a subset of CAFs with specific functions leads to poorer survival outcomes. CAFs are crucial in depositing and re-modelling the ECM within a tumour^41^. Intrinsic to this is their ability to secrete growth factors and matrix proteases, promoting and enabling tumour cell migration and invasion^85, 86^. CAFs also promote angiogenesis^87^, the proliferation and survival of tumour cells^88^, and an immunosuppressive microenvironment by reducing T cell responses^89^.

Analysing the prognostic effect of these processes in CAFs showed all were in fact correlated with a poor survival outcome. However, proteins involved in the generation and maintenance of the CAF phenotype were most prognostic with a HR approaching 3 after sensitivity analysis. This suggests that although different functional subsets of CAFs might exist, conversion of a fibroblast into a CAF is a uniting feature, creating a population of cells which ultimately contribute to poor survival outcomes in NSCLC. Targeting of this process might thus prove an effective treatment strategy. Indeed, such an approach is currently a significant area of research with a recent study showing pharmacological inhibition of NOX4, a protein important in this conversion, reduced tumour growth in mouse xenograft models^90^. In addition, a number of clinical trials targeting proteins which are also important in CAF activation such as FGFR^91^ and TGF-β^92, 93^ are currently underway with their results awaited. Other attempts at targeting molecules, such as the vitamin D receptor, which aim to revert CAFs to a more normal state are also ongoing^94^. Thus, in the case of NSCLC, the results of this meta-analysis are in keeping with treatments targeting pathways important in generating and maintaining the CAF phenotype.

Although several significant survival associations were observed in this analysis, there are a number of limitations. Some issues common in research carried out on prognostic factors^44^ have already been mentioned, such as the use of different scoring methods and cut-off values for the same marker. In addition, a number of studies did not report whether patients received neoadjuvant or adjuvant therapy and those that did failed to report sub-group outcome analyses. Since adjuvant therapy is now commonplace in treating eligible patients with lung adenocarcinoma^95^, it is feasible that CAFs could exhibit both prognostic and predictive effects. Indeed, CAFs are known to mediate increased tissue tension, a factor known to affect drug delivery^96^. Thus, future studies should include outcome measures based on therapy where possible. Similarly, several studies examined cohorts with mixed histology, generally squamous and adenocarcinoma. In this meta-analysis, there was some evidence of outcome differences between histological subtypes suggesting that subtle trends may exist, which can only be identified with subtype analysis.

A further issue with the analysis of survival data was the adjustment factors used in calculating a multivariate HR. Such adjusted values are crucial in determining the independent effects of prognostic markers^47^ but whilst many studies described these factors and the model they used, there was a significant variation in the final adjustment factors. As suggested in guidance published on reporting prognostic studies^47^, analyses could include multivariate HRs with a core, agreed set of factors alongside other models facilitating more direct comparisons for studies such as this one. In general, scoring each study against the REMARK criteria captured elements of the limitations described above, further validating the approach in conducting sensitivity analyses. Interestingly, although the REMARK criteria have been in place since 2005, there has been no increase in these scores in the intervening years. This suggests that authors should still be encouraged to comply as fully as possible with these criteria, to ensure consistent publication of high-quality studies.

Another issue with reporting of survival outcomes was that many studies published a KM plot but no associated HR. Although we used well-established methods to extract these missing values, including a novel algorithm^39^ recently published which improves upon existing methods when the number at risk is not included below the KM plot, such techniques are still associated with varying degrees of error^39, 97^. However, if no attempt is made to obtain such values, a number of studies would have been excluded and in several cases resulted in non-significant values being ignored leading to publication bias, a significant concern in any meta-analysis^47^. We assessed this using funnel plots and an asymmetry was clear in three cases and significant in one (cellular process: invasion) but this was likely due to the associated heterogeneity identified in all cases, which is another well-known cause of funnel plot asymmetry^52^. Use of extraction methods would certainly reduce publication bias this but would only apply for univariate HRs as such methods require KM plots which are not generated in a multivariate analysis. On balance, although a degree of publication bias was present in one of our random effects model, this was not the case in the remaining models and so we do not believe publication bias was prevalent in this meta-analysis.

In conclusion, the aim of this study was to address the now widely accepted hypothesis that CAFs are a heterogeneous population^41^ which is therefore likely to mean distinct functional sub-groups of CAFs represented by different proteins/markers. The study was designed in such a way as to address both of these issues by: (1) summarising the prognostic significance of every protein so far examined in CAFs in NSCLC and (2) linking each of these proteins to a cellular process that is currently believed to be crucial in CAF function. This approach is based on the fact that proteins which are prognostically important might represent key proteins that are crucial to CAF biology as well as identifying functional sub-groups within CAFs generally. An additional approach as previously mentioned is the use of scRNA sequencing experiments to identify transcriptomically-different sub-populations of CAFs. Such experiments are already yielding exciting results^22,23^ and the combination of these analyses whilst also assessing the prognostic effect of any identified proteins, as in this study, has the potential to further our understanding of CAF biology and in particular, its heterogeneity.

Notwithstanding, the current results from this study show that, despite the limitations common in prognostic research and inherent to meta-analyses, CAF expression of podoplanin or α-SMA was consistently associated with poor survival in NSCLC. Moreover, the proteins and pathways required to generate and maintain the CAF phenotype might represent potential therapeutic targets in anti-cancer treatments in NSCLC.

## Methods

This review was prospectively registered with PROSPERO (CRD42019130307), an International prospective register of systematic reviews (https://www.crd.york.ac.uk/prospero/). Guidelines for carrying out systematic reviews and meta-analysis of prognostic factor studies^47^ were followed where possible.

### Search strategy

Literature was retrieved using Medline, Embase, Scopus, Web of Science, and Cochrane databases on the 29th January, 2020 with no date restriction. All results were then updated again with a search on 24th July, 2020. The full search strategy for each database is available in Supplementary Table S6.

### Screening and selection of studies

All identified articles were exported into Rayyan^98^, a web-based application for carrying out systematic reviews. All titles, abstracts and full-text articles were independently screened by AI and SW with discrepancies resolved by consensus. The following P(atient) E(xposure) C(omparator) O(utcome), PECO was used to select articles: Patients: Individuals diagnosed with NSCLC (histological subtypes to include squamous, adenocarcinoma and large cell) who underwent surgical resection, treated with or without neoadjuvant or adjuvant therapy. Exposure: Tumour resections analysed for the presence of CAFs stained with antibodies against any protein marker using immunohistochemistry. For the definition of CAFs, an explicit statement in the methods or results section that fibroblasts, myofibroblasts, cells with a spindle-shaped morphology or similar were scored was required. Statements equivalent to positive staining within the tumour stroma or tumour microenvironment were not sufficient and such studies excluded. Comparator: Comparison of expression profiles (e.g. low/high, negative/positive) of the reported protein markers. Outcomes: The following survival outcomes were all considered for inclusion: overall survival (OS), disease-specific survival (DSS), progression-free survival (PFS), recurrence-free survival (RFS), and disease-free survival (DFS). Studies which failed to define the survival outcome were excluded.

### Data extraction

Data extraction was carried out by AI and SW with the following information for every study collected: first author; year of publication; journal; protein marker; staging, histological subtype, size and treatment details for each cohort; scoring and cut-off criteria; survival outcome, HR including associated 95% confidence intervals (CI) and *P* value. If outcome measures were related to the absence and not presence of the identified marker, the HR and associated CIs were inverted. If different studies used the same or overlapping cohorts, the largest cohort was used for the random-effects models. In the case where a KM plot was included but no associated HR was quoted, three statistical methods were used to infer the HR value.

### Methods to extract HRs from KM plots

Several methods exist to infer HRs from KM plots where they were not quoted within the article^97^. Here, we used the Parmar^42^ and Guyot^43^ methods as well as a new method, known as Nlopt^39^, based on the mathematical technique, non-linear optimisation. All three are associated with varying degrees of error^97^, but the Nlopt method is the most accurate when the number at risk (found at the bottom of a KM plot) are not included but a *P* value is; whereas the Guyot method is more accurate when the number at risk are included. The Parmar method was used when both the number at risk and *P* value were not included. All three methods rely on extracting a sufficient number of points from each KM plot. To carry this out, digitized KM plots were loaded into the Fiji distribution of Image J (version 1.52p; NIH, USA) and the axes calibrated using the Figure Calibration Plugin (Frederic V. Hessman, University of Gottingen). The specific guidance for extracting points for each method was then followed resulting in a number of X,Y points. In the case of the Parmar et al*.* method, we followed guidance from Tierney et al.^99^ to determine the minimum and maximum follow-up times for each study, as these values are crucial in extracting accurate HRs from KM plots when using this method. HRs and standard errors (SEs) for the Parmar et al*.* method were calculated in Excel, whilst we used the R script published with the Guyot et al*.* and Nlopt method to determine these HRs and SEs. The SE of the HRs were increased by 5 and 10% respectively for the Guyot/Nlopt method and Parmar method, reflecting the known error associated with each method^39, 97^.

### Study quality assessment

To assess the quality of a study, a score from the REporting recommendations for tumour MARKer prognostic studies (REMARK) criteria was calculated for each included study by AI and SW. Discrepancies were resolved by consensus. Although checklists for assessing the quality of prognostic studies do exist (e.g. the QUIPS checklist^100^), the REMARK criteria are specific to tumour marker studies and have previously been used in meta-analyses of tumour markers^101^. The REMARK criteria is composed of twenty items split into several domains: introduction, patients, specimen characteristics, assay methods, study design, statistical analysis methods, data, and analysis, and discussion. Each article was scored 1 point per item, with a score of 0.5 for items where the study fulfilled some but not all of the criteria. Cut-offs for each domain were used to represent a low-, medium- and high-quality score. For assessment of overall quality, cut-offs for low, medium and high were ≤ 10, ≤ 15 and > 15 respectively. In the case of a random effects model including a low-quality study, sensitivity analysis was used to exclude these studies and the model re-analysed. The traffic light plot in Supplementary Figure S1 was produced using the *robvis* package^102^ in R. The relationship between year of publication and REMARK score was assessed using a linear model in R and plotted using the R package *ggplot2*^103^.

### Defining the cellular processes key to CAF function

Cancer-associated fibroblasts have a wide range of functions which influence cancer progression and have been summarised in a number of recent reviews^26, 41, 48, 104–106^. These reviews were used as the basis to create a set of common cellular processes/functions crucial to CAF function (Supplementary Table S4).

### Assigning individual markers to each cellular process

To determine the proposed function of an individual CAF marker, the literature was reviewed for functional studies which investigated the role of that particular protein in some aspect of cancer progression. The following strategy was used to search Medline as a way of identifying relevant articles:

- *(name of marker) AND (cancer OR tumour OR tumor) AND (fibroblast OR stroma)*

Titles and abstracts were initially screened and the full-text reviewed if relevant. This strategy was used in preference to the alternative option of a bioinformatics approach using a database such as DAVID^107^ as the function recorded for each protein would not be specific for CAFs. Since there were only 26 identified markers, the decision to manually annotate the functional role of each marker was instead chosen as way of increasing the specificity of the highlighted functional process whilst accepting a potential loss of sensitivity. Functional studies that investigated the role of each marker in NSCLC were favoured but where these did not exist, other tumour types were used. Functional studies were occasionally determined in the same paper that also analysed the prognostic role of the particular protein in NSCLC. Functional studies generally included co-culture experiments with CAFs and tumour cells in tissue culture as well as mouse models whether these were  injection studies or genetically-engineered strains. Each marker was then placed into the relevant cellular process as identified in the Methods described above. If no relevant functional process was identified, these proteins were excluded from the analysis.

### Statistics

After extraction of all relevant data, we first combined similar survival outcomes resulting in three groups: OS/DSS, RFS/DFS and PFS. However, we considered HRs derived from either univariate or multivariate analysis separately, as recommended by Riley et al*.*^47^. In the case of an individual marker with at least 2 distinct cohorts based on the same outcome group and analysis method, a random effects model using the inverse variance method was used to create weighted HRs with 95% CIs and *P* value. A variable tree for the individual markers was generated using the *vtree* package^108^ in R. Heterogeneity was assessed by calculating *I*^2^ and Ʈ^2^ values with a *P* value generated to assess the statistical significance of the heterogeneity. The aggregate HRs for the cellular processes were calculated in the same manner but to ensure as many of the markers could be included in the analysis as possible we used HRs from the OS/DSS outcome group and combined multivariate and univariate HRs with the former used in preference to the latter where available. The ferris wheel plot was generated using *ggplot2*^103^ in R. The random effects model were carried out using the *meta* package^109^ in R. The icons representing the cellular processes in Fig. 4 are from BioRender.com. A *P* value of ≤ 0.05 was considered statistically significant for all tests carried out.

### Software

Unless otherwise stated, all analysis and figures were generated in RStudio (Version 1.3.959) with version 3.5.2 of R. Panels of figures were assembled using Adobe Illustrator 2020 (Version 24.2).

### Ethics statement

No animals or humans were used in generating data for this study.

## Supplementary Information

Supplementary Information

## Data Availability

Any of the data generated in this study are available from the corresponding author on reasonable request.
