# Supplementary material for: Characterising cancer-associated fibroblast heterogeneity in non-small cell lung cancer: a systematic review and meta-analysis
Source: Sci Rep. 2021 Feb 12;11:3727. doi: 10.1038/s41598-021-81796-2 (PMC7881148; doi:10.1038/s41598-021-81796-2)
Supplement: Supplementary file 1 — Supplementary Information [file 41598_2021_81796_MOESM1_ESM.pdf]

Supplementary File 1

Contents:

- Supplementary Table S1
- Supplementary Figure S1
- Supplementary Table S2
- Supplementary Figure S2
- Supplementary Table S3
- Supplementary Table S4
- Supplementary Table S5
- Supplementary Figure S3
- Supplementary Table S6

Characterising cancer-associated fibroblast heterogeneity in non-small cell lung cancer: a systematic review and meta-analysis

Andrew F. Irvine[1,3*], Sara Waise[1], Edward W. Green[2], Beth Stuart[4], Gareth J. Thomas[1*]

[1] School of Cancer Sciences, Faculty of Medicine, University of Southampton, Southampton, UK

[2] The German Cancer Research Centre (DKFZ), Heidelberg, Germany

[3] Current address: Department of Pathology and Data Analytics, University of Leeds, Leeds, UK

[4] Primary Care and Population Sciences, Faculty of Medicine, University of Southampton, Southampton, UK

*Correspondence:

a.f.irvine@leeds.ac.uk

or

g.thomas@soton.ac.uk

Supplementary Table S1: Summary of study characteristics of all included articles

Article ID	Author	Year	Journal	Marker	Staging & Histological Subtype	Size	Treatment (all surgical resections)	Scoring	Survival Outcome	Method used for Univariate HR	Hazard Ratios					
											Univariate			Multivariate		
											HR	95% CIs	P value	HR	95% CIs	P value
6	Koukourakis	1998	British Journal of Cancer	Thymidine Phosphorylase	T1-2, N0. Squamous (91), Adeno (50)	141	Not reported	Stromal fibroblast expression. Low: 0-50% expression; High: >50% expression	Overall Survival (OS)	Nlopt	1.09	0.58-2.05	0.80			
30	Tokunou	2001	American Journal of Pathology	c-Met	Adeno <2cm	131	Not reported	Positive when bundles of myofibroblasts were stained for c-Met in more than one microscopic area	OS	Paper	2.67	1.24-5.72	0.012	3.09	1.39-6.87	0.006
52	Swinson	2003	Journal of Clinical Oncology	Carbonic anhydrase (CAIX)	Stage I to IIIA. Large (14), squamous (109), adeno (48), other (4)	175	Adjuvant:chemotherapy (1), radiotherapy (15)	Stromal expression in myofibroblasts. Assigned score 0 to 3. 0 =negative, 1 = occasional, 2=moderate, 3=strong. 0-1 = negative; 2-3 positive	OS	Paper	1.02	0.72-1.45	0.910			
54	Koukourakis	2003	Cancer Research	SPARC	T1-2, N0-1, M0. Squamous (77), adeno (36)	113	Not reported	>50% of fibroblasts in 200x optical field exhbiting strong stromal reactivity	OS	Nlopt	2.26	1.29-3.94	0.002			
64	Ishikawa	2004	Clinical Cancer Research	MMP2	Stage I-IIIA. Squamous (77), adeno (122), other (19)	218	Adjuvant: chemotherapy (113), radiotherapy (35)	Expression in stromal fibroblast. Percentage positive cells: 0 = ≤ 25% , 1= >25%, 2= >50%. Intensity 0 = no staining, 1 = weak, 2= moderate, 3= strong. Sum of both scores. ≤3 = weak. 4,5 = strong	OS	Nlopt	1.79	1.09-2.95	0.032	1.67	0.97-2.86	0.064
90	Kawase	2008	International Journal of Cancer	Podoplanin	Staging NR.All adeno	177	Not reported	At least 10% of stromal fibroblasts showing same/higher level of expression as lymphatic endothelial cells = positive	OS	Paper	2.87	1.82-4.45	<0.001			
101	Nakao	2009	Cancer	CAIX	T1-4, N0-N2. All adeno	158	Not reported	If cell membrane and cytoplasmic staining was present in >10% CAFs, considered positive	OS	Paper	2.04	1.22-3.39	0.006	1.80	1.05-3.07	0.032
116	Kitano	2010	Archives of Pathology & Laboratory Medicine	Podoplanin	Stage I-IV. Adeno (107), squamous (70), other (4)	174	No neoadjuvant therapy	Staining: 0 = 0%, 1 = 1-50%, 2= 51-100% positive staining of myofibroblast cells. Intensity: 0= no signal, 1=weak, 2=moderate, 3=marked. Combined score of staining and intensity. Total score, 0,1=negative; 2,3 = postiive	OS	Paper				1.72	1.13-2.60	0.010
										Nlopt (Squamous & adeno)	1.66	1.06	2.61			
										Nlopt (Adeno)	2.70	1.29-5.64	0.0001			
										Nlopt (Squamous)	0.96	0.50-1.83	0.90			
151	Ito	2012	Chest	Podoplanin	Stage I. All adeno	304	Not reported	At least 10% of stromal fibroblasts showing same/higher level of expression as lymphatic endothelial cells = positive	Recurrence-free Proportion (RFP)	Paper				3.47	1.11-10.65	0.029
										Guyot	4.79	2.53-9.08	1.50E-06			
152	Neri	2012	Annals of Surgical Oncology	Podoplanin	N2 Stage disease. All adeno	112	No neoadjuvant therapy	At least 10% of stromal fibroblasts showing same/higher level of expression as lymphatic endothelial cells = positive	OS	Paper	1.022	0.64-1.64	0.927	0.77	0.47-1.29	0.309
155	Zenke	2013	Pathology International	Podoplanin	Stage I-IV. Adeno (26), squamous (19), other (7)	52	Not reported	>10% spindle-shaped cells staining for podoplanin = high	Disease-free Survival (DFS)	Paper				0.90	0.26-3.00	0.860
				CD90						Paper				1.27	0.40-3.64	0.650
157	Liao	2013	Journal of Cancer Research and Clinical Oncology	FAP	Stage I-III. Adeno (27), squamous (29), other (3)	59	No neoadjuvant therapy	8 random fields. % of staining: 0 = absent or <1%, 1 = 1-10%, 2 = 11-50%, 3>50%; intensity: 0 = none, +1 = light, +2 = moderate, +3=intense	OS	Paper				2.49	1.26-4.94	0.0087
166	Ono	2013	Chest	Podoplanin	Stage I. All squamous	142	Not reported	>= 50% of CAFs w/ positive reaction = high	OS	Parmar	2.97	1.29-6.84	0.01	2.79	1.52-5.19	0.010
									DFS	Parmar	2.64	1.33-5.24	0.005			

176	Chen	2014	Nature Communications	IGF-II	Stage I	80	No neoadjuvant therapy	IGF-II staining in CAFs. High: ≥median risk score Low: < median risk score	OS<br>Recurrence-free Survival (RFS)	Parmar<br>Parmar	3.95<br>4.51	1.24-12.64<br>1.19-17.07	0.02<br>0.03	19.15	6.32-58.00	0.0001
180	Qian	2014	American Journal of Clinical Pathology	TNFSF13	Stage I-III. Adeno(54), squamous (46), other (2)	102	No neoadjuvant therapy	Scoring in fibroblasts. Staining intensity: 0,1,2,3 = negative to strong. %, 0 = 0-29%, 1 = 20-59%, 2=60-79%, 3=80-100%. Final scoring = product of intensity + %, 0 to 1 = low expression; 2-9 = high expression	OS	Paper	1.93	1.04-3.60	0.038	2.04	1.08-3.85	0.027
181	Xing	2014	World Journal of Surgical Oncology	p-Smad2	Stage I-IIIA. Squamous (41), adeno (29), other (8)	78	Adjuvant therapy (64)	Staining in stromal fibroblasts. 10 high-power fields randomly selected. %, 0 = <5%, 1 = 5-25%, 2=26-50%, 3=>50%. Intensity, 0 = negative, 1 = weak-mod, 2= mod-strong. Two scores multiplied. Low = 0-2, high = >2	OS	Nlopt	2.15	0.95-4.90	0.049			
183	Matsuwaki	2014	Cancer Science	Podoplanin	N2 disease only. All squamous	64	Not reported	Cases where >10% of stromal fibroblasts were positive = high expression group	RFS	Nlopt	2.30	1.17-4.51	0.007	2.00	1.08-3.72	0.030
186	Koriyama	2015	Journal of Cancer Research and Clinical Oncology	Podoplanin	Post-op recurrence. All adeno	87	Adjuvant therapy	>50% of CAFs same or higher level of reactivity to endothelial cells of lymphatics	Progression-free Survival (PFS)<br>OS	Nlopt<br>Nlopt	1.80<br>1.46	1.01-3.21<br>0.82-2.59	0.028<br>0.156	1.58	0.94-2.68	0.087
188	Scherz-Shouval	2014	Cell	HSF-1	Stage I. All adeno	72	Not reported	Staining in CAFs. 0-1 = no-low-level nuclear staining, 2= intermediate, 3= high	DFS	N/A				2.20	1.30-3.80	0.005
190	Chen	2014	Tumour Biology	TGF-beta<br>alpha-SMA	Stage I-IIIA. Squamous(41), adeno (29), other (8)	78	Adjuvant: chemotherapy (64), radiotherapy (17)	Staining in stromal fibroblasts. Random selection of 10 HPF. %: 0 = <5%, 1=5-25%, 2=26-50%, 3=>50%. Intensity: 0 = negative to weak, 1 = moderate, 2= strong. For each marker, score multiplied. 0-2 = low, >2 = high	OS	Nlopt<br>Nlopt	2.33<br>2.32	1.07-5.08<br>0.97-5.53	0.019<br>0.025			
192	Kilvaer	2015	PloS One	FAP-1<br>alpha-SMA	Stage I-IIIA, squamous(289), adeno (201), other (46)	536	No neoadjuvant therapy	0 = no staining, 1 = 1–10%, 2 = 11–50% and 3 = > 50%. High expression defined as optimal cut-off points for survival. Fap = >0.5, a-SMA = >2	DSS	Paper<br>Paper	0.75<br>0.93	0.54-1.03<br>0.7-1.23	0.070<br>0.597			
202	Neri	2015	International Journal of Cancer	Podoplanin	Tumour sizes 3-5cm, N0	78	Not reported	Spindle-shaped cells in the cancer stroma. High: ≥median risk score Low: < median risk score	DSS	Nlopt	3.38	1.37-8.31	0.012			
203	Yoshida	2015	Clinical Cancer Research	Podoplanin	Post-op recurrence. All adeno	106	Treated with EGFR-TKIs as 1st line chemotherapy	At least 10% of stromal fibroblasts showing same/higher level of expression as lymphatic endothelial cells = positive	PFS	Paper	1.76	1.15-2.71	0.010	2.00	1.19-3.42	0.010
219	Chen	2016	Oncotarget	HGF	Stage I-IIIA. Squamous (56), adeno (65), Other (13)	134	Adjuvant: cisplatin (112), radiotherapy (5)	Staining in stromal fibroblasts. %: 0 = <5%, 1 = 5-25%, 2 = 26-50%, 3= >50%. Intensity: 0 = neg-weak, 1=weak-mod, 2=mod-strong. Scores multiplied. 0-2 = low, >2 = high	OS	Paper	2.36	1.44-3.88	0.001	1.89	1.12-3.20	0.017
221	Nitsche	2016	Oncology Letters	alpha-SMA<br>Periostin	T1-T3, N0-N3. Adeno(66), squamous (22), other (5)	93	Not reported	Staining in myofibroblasts. Scanned slides analysed for total surface area vs stained area. Median values to define low and high for a-SMA	OS	Paper<br>Paper	1.03<br>1.8	0.56-1.88<br>0.99-3.27	0.93<br>0.05	 1.92	 0.81-4.76	 0.137

228	Ishibashi	2017	Scientific Reports	CD200	Recurrence after gefitinib treatment. All adeno	90	Patients with EGFR-activating mutations treated with gefitinib	Spindle-shaped cells within the stroma with at least 5% of these fibroblasts positive for CD200	PFS<br>RFS	Nlopt<br>Nlopt	0.63<br>0.77	0.40-0.98<br>0.502-1.184	0.057<br>0.249			
229	Shimizu	2017	Journal of Cancer Research and Clinical Oncology	Cav-1	Stage I, R0. All adeno	412	Not reported	>50% of stromal fibroblasts showed strong staining = positive	PFS<br>OS	Guyot<br>Guyot	0.34<br>0.36	0.21-0.53<br>0.19-0.68	4.10E-06<br>0.0016	0.75	0.45-1.25	0.226
243	Yurugi	2017	Anticancer Research	Podoplanin	Stage IA-IIIA. All squamous	126	No neoadjuvant therapy	>10% of CAFs showed immunoreactivity equal or greater than lymphatic endothelium/type I pneuomocytes. CAFs defined as spindle-shaped cells present in tumour stroma	DSS<br>DFS	Nlopt<br>Nlopt	2.25<br>2.09	1.16-4.32<br>1.15-3.78	0.006<br>0.006	2.17<br>2.08	1.09-4.32<br>1.09-3.96	0.027<br>0.027
248	Nakamura	2017	Lung Cancer	CAIX	Tumour >3cm. All adeno	188	No neoadjuvant or adjuvant therapy	If cell membrane/cytoplasm in >10% of CAFs = positive	RFS	Guyot	1.8	1.19-2.72	0.005	1.63	1.07-2.48	<0.05
249	Luo	2018	Cellular Physiology and Biochemistry	alpha-SMA	Stage IA-VA. Adeno (68), other (14)	82	Not reported	Staining in CAFs. An IHC score of >= 3 (++, +++) defined as over-expression of a-SMA	OS<br>RFS	Paper<br>Paper	7.64<br>7.73	1.74-33.46<br>2.26-26.46	0.007<br>0.001	5.97<br>4.02	0.71-50.13<br>0.89-18.11	0.100<br>0.07
252	Chen	2018	Journal of Clinical Pathology	FAP	Stage I-III. All squamous	122	No neoadjuvant therapy	Histological evaulation of CAFs. Intensity: Grade 0 = absent or <1%, 1 = 1-10%, 2=11-50%, 3= >50%. High expression, grade >2	OS<br>DFS	Paper<br>Paper	2.56<br>2.5	1.51-4.36<br>1.47-4.24	0.001<br>0.001	2.03<br>1.99	1.03-3.99<br>1.02-6.82	0.04<br>0.047
257	Ji	2018	Medicine	CXCL14	Stage I-IIIA. Squamous (44), adeno (58), other (4)	106	Adjuvant: chemotherapy (67), radiotherapy (37)	Staining in stromal fibroblasts. Intensity: 0 = absent, 1= weak, 2= moderate, 3= strong. %: 0=no staining, 1=1-5%, 2=5-25%, 3=25-50%, 4=50-75%, 5=75-100%. Multiplied. Low = 0-8, high = 9-15	OS<br>PFS	Nlopt<br>Nlopt	5.64<br>4.71	2.87-11.06<br>2.75-8.06	<0.001<br><0.001	2.63<br>3.26	1.01-6.82<br>1.71-6.07	0.047<br><0.001
260	Zhang	2018	Cancer Research	GFAT-2	Stage I-IV. All adenocarcinoma	211	Not reported	Staining in fibroblasts. Qualitative ordinal scoring: 0 =negative, 1 = low, 2=moderate, 3=high. Low group = 0+1, high group = 2+3	OS	Paper	2.05	1.2-3.11	0.006	1.30	1.07-1.57	0.007
261	Sakai	2018	Lung Cancer	Podoplanin	Stage I. All adenocarcinoma	174	No neoadjuvant therapy	Spindle cells in stroma identified as CAFs. Neg = <10%, positive = >10%. Grade 0 = CAF area/stromal area <10%, 1 = 10-50%, 2 = >50%	RFS	Paper	2.48	1.07-5.54	0.029	1.30	0.53-3.07	0.560
263	Onion	2018	Oncotarget	Cav-1<br>Tn-C	Stage I-III	62	Not reported	Staining of stromal fibroblasts. Degree of staining: 0 = 0-1%, 1=2-10%, 2=11-50%, 3= >= 50%; low=0-1, high=2-3	OS	Paper				1.57<br>2.31	1.57-2.42<br>1.29-4.14	0.038<br>0.005
265	Kubouchi	2018	Histopathology	Podoplanin	Stage IA. All adenocarcinoma	158	No neoadjuvant therapy	Spindle cells in cancer stroma identified as CAFs. At least 10% of stromal fibroblasts showing same/higher level of expression as lymphatic endothelial cells = positive	DSS<br>DFS	Paper<br>Paper	2.06<br>2.73	1.09-3.88<br>1.75-4.26	0.025<br><0.001	2.07<br>4.41	0.50-8.62<br>1.62-12.05	0.316<br>0.004
266	Nakasone	2018	Journal of Cancer Research and Clinical Oncology	Podoplanin	Tumour >3cm. All adeno	97	Not reported	At least 10% of spindle cells stained positive within tumour stroma = positive	OS<br>RFS	Guyot<br>Guyot	1.64<br>1.83	0.86-3.13<br>1.04-3.23	0.132<br>0.036			
271	Kilvaer	2019	PloS One	PDGFR-alpha<br>PDGFR-beta	Stage I-IIIA. Squamous (273), adeno (187), Other (42)	502	No neoadjuvant therapy	0 = no staining, 1 = 1–10%, 2 = 11–50% and 3 = > 50%. High expression defined as optimal cut-off points for survival.PDGFR-a = >1.75, PDGFR-b = >1.5	DSS<br>DSS	Paper<br>Paper	0.77<br>1.22	0.56-1.06<br>0.92-1.61	0.105<br>0.169			

273	Qiu	2019	Thoracic Cancer	alpha-SMA	Stage I-IIIA. All adenocarcinoma	208	No neoadjuvant therapy	Staining in CAFs. 4 high-powered fields in tumour stroma randomly selected. Intensity: negative = 0, weak = 1, moderate = 2, strong = 3. %: 0% = 0, 1-10% = 1, 11-50% = 2, >50% = 3. 0-2 = negative; >2 = positive	OS	Paper	7.03	4.27-11.57	<0.001	4.78	2.78-8.23	<0.001
									RFS	Paper	8.19	5.11-13.12	<0.001	5.56	3.36-9.20	<0.001
281	Nowinska	2019	Cancers	Irsin	Stage I-IV. Squamous (381), adeno (348)	729	Not reported	Staining in stromal fibroblasts. Intensity: 1 - weak, 2- moderate, 3- strong; % 0 - no expression; 1 - 1-10%, 2 - 11-50%, 3- 51-80%, 4 - >80%. Final result was product of the two. ≤4 = low, >4 = high	OS	Paper	1.33	1.08-1.63	0.0054	1.30	1.06-1.60	0.011
284	Schuzle	2019	Thoracic Cancer	alpha-SMA	Stage I-III. Adeno (112), squamous (146), large (46)	304	Neoadjuvant chemo/ chemo-radiotherapy (90). Adjuvant: chemotherapy (13), radiotherapy (53)	Proportional amount of CD34+ and a-SMA+ fibroblasts from tumour stroma recorded. Positive CD34 = >= 1% cells. a-SMA = >= 20% cells.	OS	Nlopt	1.29	0.96-1.75	0.077			
									PFS	Nlopt	1.35	1.00-1.83	0.042			
				CD34					OS	Nlopt	0.72	0.54-0.95	0.024	0.72	0.53-0.96	0.028
									PFS	Nlopt	0.66	0.50-0.89	0.008	0.60	0.44-0.82	0.001
285	Inoue	2019	Virchows Archiv	alpha-SMA	Stage I-IV. All adeno	92	No neoadjuvant therapy	Staining in fibroblast-like stroma cells. Median values of positive areas to total stromal area were a-SMA>= 60% high, podo >=1% high, periostin >= 40% high	OS	Nlopt	3.91	1.68-9.10	0.0044			
				Podoplanin							2.89	1.32-6.32	0.01			
				Periostin							1.13	0.50-2.57	0.768			
288	Alcaraz	2019	Lung Cancer	alpha-SMA	Stage IA-IVA, adeno (105), squamous (92), large (12)	208	Not reported	Positive fraction in TAFs calculated with binary mask. Thresholds calculated by maximising Youden's index *J*.	OS	Nlopt	1.58	1.04-2.41	0.044	1.89	1.18-3.04	0.009
289	Saito	2010	Cancer Research	Forkhead box F1 (FoxF1)	Stage I-IIIA	247	No neo- or adjuvant-therapy	Nuclear staining of FoxF1 in cells identified as stromal spindle cells. Positivity of FoxF1 in CAFs scored as positive.	OS	Nlopt	1.08	0.83-1.42	0.571			

Supplementary Figure S1

Quality Assessment Domains

Study	Introduction	Patients	Specimens	Assay	Study Design	Statistics	Data	Analysis	Discussion	Overall
Koukourakis 1998	+	+	+	+	+	+	X	X	+	−
Tokunou 2001	+	−	+	+	+	−	−	+	+	−
Swinson 2003	+	+	+	+	+	+	−	+	+	+
Koukourakis 2003	+	+	+	+	+	+	X	+	+	−
Ishikawa 2004	+	+	+	+	−	+	X	+	+	−
Kawase 2008	+	+	+	+	+	+	X	+	+	+
Nakao 2009	+	+	+	+	+	+	X	+	+	+
Kitano 2010	+	+	+	+	+	+	−	+	+	+
Ito 2012	+	+	+	+	+	+	X	−	+	−
Neri 2012	+	+	+	+	+	+	X	+	+	+
Zenke 2013	+	+	+	+	+	+	−	−	+	+
Liao 2013	+	+	+	+	+	+	−	−	+	−
Ono 2013	+	−	+	+	+	+	−	−	+	−
Chen 2014	+	+	+	+	+	+	-	+	+	+
Qian 2014	+	+	+	+	+	+	X	+	+	−
Xing 2014	+	+	+	+	+	+	X	−	+	−
Matsuwaki 2014	+	−	+	+	+	+	X	−	+	−
Koriyama 2015	+	+	+	+	+	+	−	−	+	−
Scherz−Shouval 2014	+	X	+	+	+	+	X	−	+	−
Kilvaer 2015	+	+	+	+	+	+	−	+	+	+
Neri 2015	+	−	+	+	+	+	X	X	+	X
Yoshida 2015	+	−	+	+	+	+	−	+	+	−
Chen 2016	+	−	+	+	+	+	−	+	+	+
Nitsche 2016	+	−	+	+	+	+	−	+	+	−
Ishibashi 2017	+	−	+	+	+	+	−	−	+	−
Shimizu 2017	+	−	+	+	+	+	−	−	+	−
Yurugi 2017	+	+	+	+	+	+	−	−	+	+
Nakamura 2017	+	+	+	+	X	+	−	+	+	−
Luo 2018	+	−	+	+	−	+	X	−	+	−
Chen 2018	+	+	+	+	+	+	−	+	+	+
Ji 2018	+	+	+	+	+	+	−	−	+	+
Zhang 2018	+	+	+	+	+	+	−	+	+	+
Sakai 2018	+	+	+	+	+	+	−	+	+	+
Onion 2018	+	X	+	+	X	+	X	−	+	X
Kubouchi 2018	+	+	+	+	+	−	−	+	+	+
Nakasone 2018	+	−	+	+	+	+	−	−	+	−
Kilvaer 2018	+	+	+	+	−	+	+	+	+	+
Qiu 2019	+	+	+	+	X	+	−	+	+	−
Nowinska 2019	+	X	+	+	+	+	−	+	+	−
Schulze 2019	+	+	+	+	+	+	−	+	+	+
Inoue 2019	+	+	+	+	−	+	−	−	+	−
Alcaraz 2019	+	−	+	+	+	+	−	+	+	−
Saito 2010	+	+	+	+	+	−	X	X	+	X

Quality Assessment

- + High
- − Medium
- X Low

Supplementary Figure 1: Quality assessment scores calculated for each study against the REMARK criteria. Cut-offs for each domain were used to assign scores a quality rating of low, medium or high. The cut-offs for the overall scores for low, medium and high were: ≤10, ≤15, >15, respectively.

Supplementary Table S2: Scores of each included study based on the REMARK criteria

Article Details			Introduction	Materials and Methods								Results									Discussion		Overall (/20)
Author	*Article ID*	*Year*		*Patients*		*Specimen Characteristics*	*Assay*	*Study design*				*Statistics*		*Data*		*Analysis & Presentation*							
			1	2	3	4	5	6	7	8	9	10	11	12	13	14	15	16	17	18	19	20	
Koukourakis	6	1998	1	1	1	1	1	1	0.5	1	0	1	1	0	0	0	0	0	0	0	1	1	11.5
Tokunou	30	2001	1	0.5	1	1	1	1	0	1	0	0.5	0.5	0	1	1	1	1	1	0	1	1	14.5
Swinson	52	2003	1	1	1	1	1	1	0	1	0	1	1	1	0	0	1	0	1	1	1	1	15
Koukourakis	54	2003	1	1	1	1	1	1	0.5	1	0	1	1	0	0	0	1	1	1	0	1	1	14.5
Ishikawa	64	2004	1	1	1	1	1	0.5	0	1	0	1	1	0	0	1	0	1	1	0	1	1	13.5
Kawase	90	2008	1	1	1	1	1	1	1	1	0	1	1	0	0	1	1	0	1	0	1	1	15
Nakao	101	2009	1	1	1	1	1	1	1	1	0	1	1	0	0	1	1	0.5	1	0	1	1	15.5
Kitano	116	2010	1	1	1	1	1	0.5	0.5	1	0	1	1	0	1	1	0	1	1	0	1	1	15
Ito	151	2012	1	1	1	1	1	1	1	1	0	1	1	0	0	1	0	0.5	1	0	1	1	14.5
Neri	152	2012	1	1	1	1	1	1	1	1	0	1	1	0	0	1	1	1	1	0	1	1	16
Zenke	155	2013	1	1	1	1	1	1	1	1	0	1	1	0	1	0	0	1	1	0	1	1	15
Liao	157	2013	1	1	1	1	1	1	1	1	0	1	1	0	1	0	0	0.5	0.5	0	1	1	14
Ono	166	2013	1	1	0	1	1	1	0.5	1	0	1	1	0	1	0	0	0.5	1	0	1	1	13
Chen	176	2014	1	1	1	1	1	0.5	0.5	1	0	1	1	0	0	1	0	1	1	0	1	1	14
Qian	180	2014	1	1	1	1	1	0.5	0.5	1	0	1	1	0	0	1	1	0.5	1	0	1	1	14.5
Xing	181	2014	1	1	1	1	1	1	1	1	0	1	1	0	0	1	0	0	0	0	1	1	13
Matsuwaki	183	2014	1	1	0	1	1	1	1	1	0	1	1	0	0	0	0	0.5	1	0	1	1	12.5
Koriyama	186	2015	1	1	1	1	1	0.5	1	1	0	1	1	0	1	0	0	0.5	1	0	1	1	14
Scherz-Shouval	188	2014	1	0	0	1	1	1	0.5	1	0	1	1	0	0	0	0	1	1	0	1	1	11.5
Chen	190	2014	1	1	1	1	1	1	1	1	0	1	1	0	1	1	0	0	1	0	1	1	15
Kilvaer	192	2015	1	1	1	1	1	1	1	1	0	1	1	0	1	1	1	1	1	0	1	1	17
Neri	202	2015	1	0.5	0	1	1	0	1	1	0	1	1	0	0	0	0	0	0	0	1	1	9.5
Yoshida	203	2015	1	0	1	1	1	0	1	1	0	1	1	0	1	1	1	0.5	1	0	1	1	14.5
Chen	219	2016	1	0.5	0.5	1	1	1	1	1	0	1	1	0	1	1	1	0.5	1	0	1	1	15.5
Nitsche	221	2016	1	1	0	1	1	0	1	1	0	1	1	0	1	0	1	1	1	0	1	1	14
Ishibashi	228	2017	1	0.5	1	1	1	0	1	1	0	1	1	0	1	1	0	0	0	0	1	1	12.5
Shimizu	229	2017	1	1	0	1	1	0	1	1	0	1	1	0	1	1	0	0.5	1	0	1	1	13.5
Yurugi	243	2017	1	1	1	1	1	1	1	1	0	1	1	0	1	1	0	0.5	1	0	1	1	15.5
Nakamura	248	2017	1	1	1	1	1	0	0	1	0	1	1	0	1	1	0	1	1	0	1	1	14
Luo	249	2018	1	0.5	0	1	1	0	0.5	1	0	1	1	0	0	0	1	0.5	1	0	1	1	11.5
Chen	252	2018	1	1	1	1	1	1	1	1	0	1	1	0	1	1	1	0.5	1	0	1	1	16.5
Ji	257	2018	1	1	1	1	1	1	1	1	0	1	1	0	1	1	0	0.5	1	0	1	1	15.5
Zhang	260	2018	1	1	1	1	1	1	1	1	0	1	1	0	1	1	1	1	1	0	1	1	17
Sakai	261	2018	1	1	1	1	1	1	0	1	0	1	1	0	1	1	1	0.5	1	0	1	1	15.5
Onion	263	2018	1	0	0	1	1	0	0	1	0	1	1	0	0	0	0	1	1	0	1	1	10
Kubouchi	265	2018	1	1	1	1	1	1	1	1	0	0	1	0	1	1	1	0.5	1	0	1	1	15.5
Nakasone	266	2018	1	0.5	0	1	1	0	1	1	0	1	1	0	1	1	0	0	0	0	1	1	11.5
Kilvaer	271	2018	1	1	1	1	1	0	0.5	1	0	1	1	1	1	1	1	0.5	1	0	1	1	16
Qiu	273	2019	1	1	1	1	1	0	0	1	0	1	1	0	1	1	1	0.5	1	0	1	1	14.5
Nowinska	281	2019	1	0	0	1	1	0	1	1	0	1	1	0	1	1	1	0.5	1	0	1	1	13.5
Schulze	284	2019	1	1	1	1	1	1	1	1	0	1	1	0	1	1	0	1	1	0	1	1	16
Inoue	285	2019	1	1	1	1	1	0	0.5	1	0	1	1	0	1	1	0	0	0	0	1	1	12.5
Alcaraz	288	2019	1	1	0	1	1	0.5	1	1	0	1	1	0	1	1	0	1	1	0	1	1	14.5
Saito	289	2010	1	1	1	1	1	1	0	1	0	1	0	0	0	0	0	0	0	0	1	1	10

Supplementary Figure S2

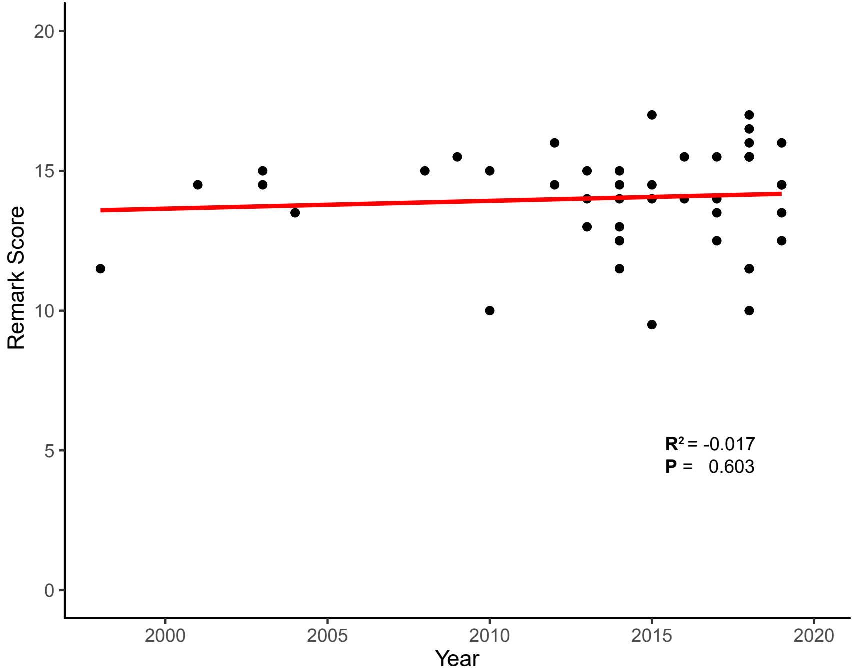

Supplementary Figure S2: X,Y plot of Year vs Remark score for each included study. Red line represents fit of a linear relationship between Year and Remark score.

Supplementary Table S3

Marker/Cellular Process	Outcome	Analysis	Histology	Studies	Random Effects Model		Heterogeneity		
					Overall Effect (95% CIs)	P value	I^2 (%)	τ^2	P value
Podoplanin	OS/DSS	U	All	7	2.20 (1.75-2.77)	<0.001	0	0.00	0.64
			Adeno	5	2.42 (1.83-3.21)	<0.001	0	0.00	0.68
Generation & maintenance of CAF phenotype	OS/DSS	M	All	5	2.74 (1.74-4.33)	<0.001	45.8	0.01	0.12
Re-modelling of extracellular matrix	OS/DSS	M	All	9	1.82 (1.45-2.15)	<0.001	45	0.08	0.07
Immunosupp ression	OS/DSS	M	All	5	1.68 (1.03-2.73)	0.040	66.7	0.19	0.02
Promoting tumour angiogenesis	OS/DSS	M	All	3	1.56 (1.02-2.39)	0.040	14.5	0.02	0.31
Proliferation and survival of tumour cells	OS/DSS	M	All	6	2.34 (1.12-4.88)	0.020	87.40	0.49	<0.01

Supplementary Table S3: Sensitivity analysis excluding articles with low remark scores. Random effects model scores.

Supplementary Table S4: Consenus Functions of CAFs

**CAF Function**
1. Generation and maintenance of CAF phenotype
2. Re-modelling of extracellular matrix
3. Immunosuppression
4. Promoting migration and invasion of tumour cells
5. Promoting tumour angiogenesis
6. Proliferation and survival of tumour cells
7. Metabolic regulation of tumour cells

Supplementary Table S4: Key Functions Identified in CAFs Required for Tumour Progression

Supplementary Table S5

Protein Marker	Uniprot ID	Gene Name	Biological Process	Comments
α-SMA	P62736	ACTA2	Regulation of cytoskeleton and contractility of fibroblasts [1]	Mechano-signalling is known to be required for the generation and maintenance of CAFs[2]
Carbonic anhydrase IX	Q16790	CA9	Response to hypoxia (tumour metabolism) [3]	
Caveolin-1	Q03135	Cav1	Tumour angiogenesis, cell proliferation [4]	Down-regulation of cav-1
CD200 (Ox-2)	P41217	CD200	Augments anti-tumour effect of gefitinib, triggers immunosuppressive response [5]	
CD90 (Thy-1)	P04216	Thy1	Contractility-dependent mechano-signalling [6,7]	Mechano-signalling is known to be required for the generation and maintenance of CAFs[2]
CD34	P28906	CD34	No clear role in fibroblasts currently [8]	First study to analyse role of CD34 in CAFs.
c-MET (HGFR)	P08581	MET	Promoting invasion and metastasis [9]	Original paper
CXCL14	O95715	CXCL14	Promotion of tumour growth, promoting angiogenesis, enhancing cell migration and invasion of cancer cells [10-12]	
FAP	Q12884	FAP	Re-modelling of ECM, enhancing cell migration of cancer cells [13]	
GFAT2	O94808	GFAT2	Metabolic regulation of tumour cells [14]	Original paper
HGF	P14210	HGF	Enhances cell migration/invasion of cancer cells, enhances cell survival and tumour progression [15,16]	
HSF-1	Q00613	HSF1	Angiogenesis, re-modelling of ECM, cell migration/invasion [17]	Original paper
IGF-II	P01344	IGF2	Enhancing proliferation/survival of cancer cells [18,19]	Original paper
Irisin (FNDC5)	Q8NAU1	FNDC5	No established role in fibroblasts currently [20]	First study to analyse role of Irisin in CAFs.

MMP2	P08253	MMP2	Angiogenesis, re-modelling of ECM, cell migration/invasion [21]	Original paper
PDGFR-α	P16234	Pdgfra	Enhancing proliferation/survival of cancer cells [22,23]	
PDGFR-β	P09619	Pdgfrb	Cell migration/invasion [24,25]	
Periostin	Q15063	Postn	Cell migration/invasion, enhancing proliferation/cell survival [26-29]	Role of periostin in TME reviewed in [30]
Podoplanin	Q86YL7	PDPN	Cell migration/invasion, immunosuppressive microenvironment, re-modelling of ECM [31-36]	
p-Smad2	Q15796	Smad2	Activation of CAFs via TGF-beta pathway [37]	Original paper
SPARC	P09486	SPARC	Remodelling ECM [38,39]	Original paper
Tenascin-C	P24821	Tnc	Negative regulation of immune surveillance, ECM remodelling [40,41]	
TGF-β	P01137	TGFB1	Activation of CAFs via TGF-beta pathway [42]	Original paper
Thymidine Phosphorylase	P19971	TYMP	Angiogenesis [43]	Original paper
TNFSF13 (April)	O75888	TNFSF13	Cell proliferation [44]	
Forkhead fox F1 (FoxF1)	Q12946	FOXF1	Generation and maintenance of CAF phenotype [45]	Original paper

Supplementary Table S5: Functions of each of the 26 protein markers were identified using a literature search in Medline (see Methods for more detail).

Supplementary Figure S3

Funnel Plots

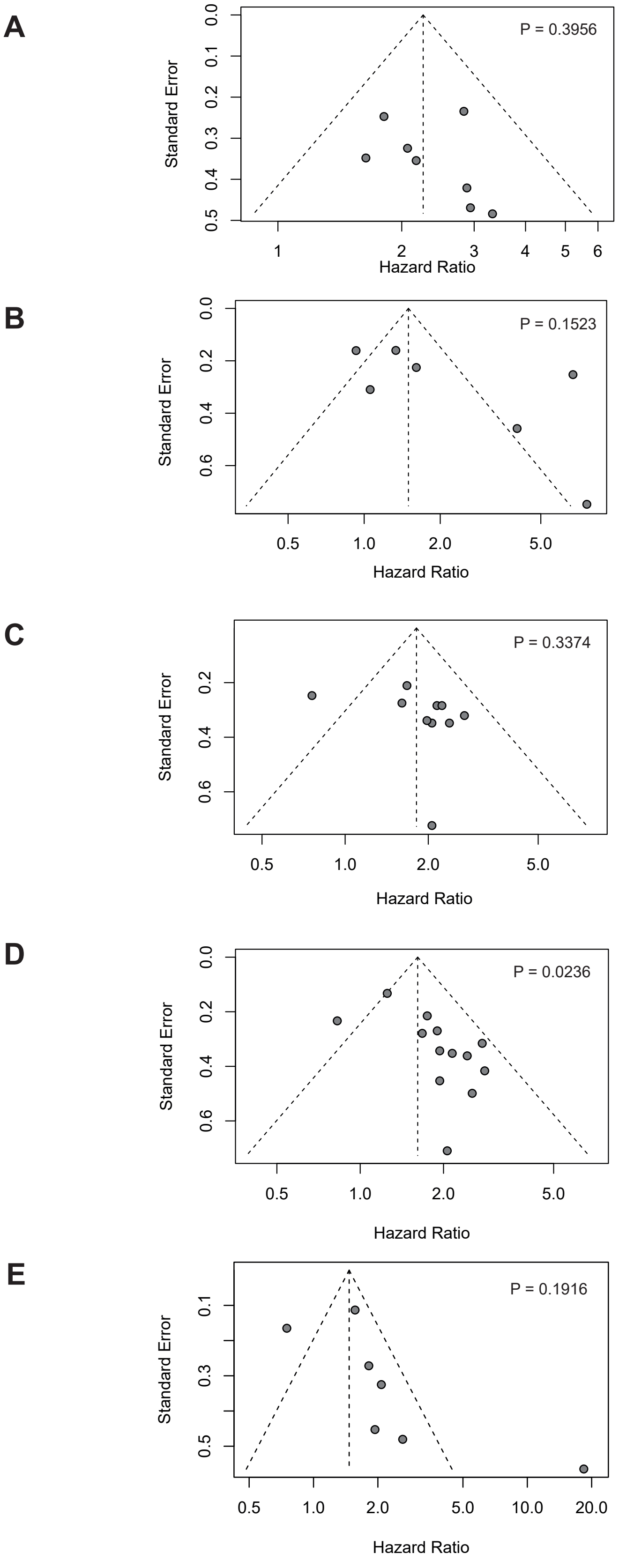

Supplementary Figure S3: Funnel plots of meta-analyses with n ≥ 7 studies.
(**A**) Podoplanin > OS/DSS > Univariate analysis, **B**) α-SMA > OS/DSS > Univariate analysis, (**C**) Cellular processes: ECM, **D**) Cellular processes: Invasion, **E)** Cellular processes: Proliferation

Supplementary Table S6

Embase/MEDLINE search (Ovid)

Ovid MEDLINE(R) and Epub Ahead of Print, In-Process & Other Non-Indexed Citations and Daily 1946 to 15th July, 2020

Embase Classic+Embase 1947 to 15th July, 2020

#	Search
1	survival*.mp. [mp=title, abstract, heading word, drug trade name, original title, device manufacturer, drug manufacturer, device trade name, keyword, floating subheading word, candidate term word]
2	(IHC or immunohistochem* or staining or biomarker*).mp. [mp=title, abstract, heading word, drug trade name, original title, device manufacturer, drug manufacturer, device trade name, keyword, floating subheading word, candidate term word]
3	(fibroblast* or myofibroblast* or stroma*).mp. [mp=title, abstract, heading word, drug trade name, original title, device manufacturer, drug manufacturer, device trade name, keyword, floating subheading word, candidate term word]
4	(cancer* or tumor* or tumour* or carcinoma* or metasta* or adenocarcinoma*).mp. [mp=title, abstract, heading word, drug trade name, original title, device manufacturer, drug manufacturer, device trade name, keyword, floating subheading word, candidate term word]<br>5. (lung* or NSCLC or SCLC).mp. [mp=title, abstract, heading word, drug trade name, original title, device manufacturer, drug manufacturer, device trade name, keyword, floating subheading word, candidate term word]
5	(lung* or NSCLC or SCLC).mp. [mp=title, abstract, heading word, drug trade name, original title, device manufacturer, drug manufacturer, device trade name, keyword, floating subheading word, candidate term word]
6	(prognos* or predict*).mp. [mp=title, abstract, heading word, drug trade name, original title, device manufacturer, drug manufacturer, device trade name, keyword, floating subheading word, candidate term word]
7	1 or 2 or 6
8	3 and 4 and 5 and 7
9	limit 8 to (embase or medline)

Cochrane search

#	Search
1	prognos*:ti,ab,kw
2	survival*:ti,ab,kw
3	IHC or immunhistochem* or staining* biomarker* OR predict*:ti,ab,kw
4	1 OR 2 OR 3
5	(fibroblast* or myofibroblast* or stroma*):ti,ab,kw
6	(cancer* or tumour* or tumor* or carcinoma* or metasta* or adenocarcinoma*):ti,ab,kw
7	(lung* OR NSCLC OR SCLC):ti,ab,kw
8	#4 AND #5 AND #6 AND #7

Scopus Search

#	Search
1	(TITLE-ABS-KEY(survival*) OR TITLE-ABS-KEY(IHC OR immunohistochem* OR staining OR biomarker*) OR TITLE-ABS-KEY(prognos* OR predict*))
	AND
2	(TITLE-ABS-KEY(fibroblast* OR myofibroblast* OR stroma*))
	AND
3	(TITLE-ABS-KEY(cancer* OR tumor* OR tumour OR carcinoma* OR metasta*))
	AND
4	(TITLE-ABS-KEY(lung* OR NSCLC OR SCLC)) AND (EXCLUDE (DOCTYPE,"cp"))

Web of science Search

#	Search
1	TS=survival*
2	TS=(IHC OR immunohistochem* OR staining OR biomarker*)
3	TS=(prognos* OR predict*)
4	TS=(fibroblast* OR myofibroblast* OR stroma*)
5	TS=(cancer* OR tumor* OR tumour* OR carcinoma* OR metasta* OR adenocarcinoma)
6	TS=(lung* OR NSCLC OR SCLC)
7	#3 OR #2 OR #1
8	#7 AND #6 AND #5 AND #4

Supplementary Table S6: Search criteria used for each database. Searches were initially carried out on 29/01/2020 but re-run on 24/07/2020.

Supplementary File References

1 Nurmik, M., Ullmann, P., Rodriguez, F., Haan, S. & Letellier, E. In search of definitions: Cancer-associated fibroblasts and their markers. *Int J Cancer* **146**, 895-905, doi:10.1002/ijc.32193 (2020).

2 Calvo, F. *et al.* Mechanotransduction and YAP-dependent matrix remodelling is required for the generation and maintenance of cancer-associated fibroblasts. *Nat Cell Biol* **15**, 637-646, doi:10.1038/ncb2756 (2013).

3 Fiaschi, T. *et al.* Carbonic anhydrase IX from cancer-associated fibroblasts drives epithelial-mesenchymal transition in prostate carcinoma cells. *Cell Cycle* **12**, 1791-1801, doi:10.4161/cc.24902 (2013).

4 Chen, D. & Che, G. Value of caveolin-1 in cancer progression and prognosis: Emphasis on cancer-associated fibroblasts, human cancer cells and mechanism of caveolin-1 expression (Review). *Oncol Lett* **8**, 1409-1421, doi:10.3892/ol.2014.2385 (2014).

5 Ishibashi, M. *et al.* CD200-positive cancer associated fibroblasts augment the sensitivity of Epidermal Growth Factor Receptor mutation-positive lung adenocarcinomas to EGFR Tyrosine kinase inhibitors. *Scientific Reports* **7**, 46662 (2017).

6 Barker, T. H. & Hagood, J. S. Getting a grip on Thy-1 signaling. *Biochim Biophys Acta* **1793**, 921-923, doi:10.1016/j.bbamcr.2008.10.004 (2009).

7 Fiore, V. F., Ju, L., Chen, Y., Zhu, C. & Barker, T. H. Dynamic catch of a Thy-1-α5β1+syndecan-4 trimolecular complex. *Nat Commun* **5**, 4886, doi:10.1038/ncomms5886 (2014).

8 Schulze, A. B. *et al.* Prognostic impact of CD34 and SMA in cancer-associated fibroblasts in stage I-III NSCLC. *Thorac Cancer* **11**, 120-129, doi:10.1111/1759-7714.13248 (2020).

9 Tokunou, M. *et al.* c-MET expression in myofibroblasts: Role in autocrine activation and prognostic significance in lung adenocarcinoma. *American Journal of Pathology* **158**, 1451-1463 (2001).

10 Augsten, M. *et al.* Cancer-associated fibroblasts expressing CXCL14 rely upon NOS1-derived nitric oxide signaling for their tumor-supporting properties. *Cancer Res* **74**, 2999-3010, doi:10.1158/0008-5472.can-13-2740 (2014).

11 Augsten, M. *et al.* CXCL14 is an autocrine growth factor for fibroblasts and acts as a multi-modal stimulator of prostate tumor growth. *Proc Natl Acad Sci U S A* **106**, 3414-3419, doi:10.1073/pnas.0813144106 (2009).

12 Sjöberg, E. *et al.* A Novel ACKR2-Dependent Role of Fibroblast-Derived CXCL14 in Epithelial-to-Mesenchymal Transition and Metastasis of Breast Cancer. *Clin Cancer Res* **25**, 3702-3717, doi:10.1158/1078-0432.Ccr-18-1294 (2019).

13 Lee, H. O. *et al.* FAP-overexpressing fibroblasts produce an extracellular matrix that enhances invasive velocity and directionality of pancreatic cancer cells. *BMC Cancer* **11**, 245, doi:10.1186/1471-2407-11-245 (2011).

14 Zhang, W. *et al.* GFPT2-expressing cancer-associated fibroblasts mediate metabolic reprogramming in human lung adenocarcinoma. *Cancer Research* **78**, 3445-3457 (2018).

15 Ding, X. *et al.* HGF-mediated crosstalk between cancer-associated fibroblasts and MET-unamplified gastric cancer cells activates coordinated tumorigenesis and metastasis. *Cell Death Dis* **9**, 867, doi:10.1038/s41419-018-0922-1 (2018).

16 Kanaji, N. *et al.* Hepatocyte growth factor produced in lung fibroblasts enhances non-small cell lung cancer cell survival and tumor progression. *Respir Res* **18**, 118, doi:10.1186/s12931-017-0604-z (2017).

17 Scherz-Shouval, R. *et al.* The reprogramming of tumor stroma by HSF1 is a potent enabler of malignancy. *Cell* **158**, 564-578 (2014).

18 Cacheux, W. *et al.* Interaction between IGF2-PI3K axis and cancer-associated-fibroblasts promotes anal squamous carcinogenesis. *Int J Cancer* **145**, 1852-1859, doi:10.1002/ijc.32178 (2019).
19 Chen, W. J. *et al.* Cancer-associated fibroblasts regulate the plasticity of lung cancer stemness via paracrine signalling. *Nature Communications* **5** (2014).
20 Nowinska, K. *et al.* Expression of Irisin/FNDC5 in Cancer Cells and Stromal Fibroblasts of Non-small Cell Lung Cancer. *Cancers (Basel)* **11**, doi:10.3390/cancers11101538 (2019).
21 Ishikawa, S. *et al.* Matrix metalloproteinase-2 status in stromal fibroblasts, not in tumor cells, is a significant prognostic factor in non-small-cell lung cancer. *Clinical Cancer Research* **10**, 6579-6585 (2004).
22 Gerber, D. E. *et al.* Stromal platelet-derived growth factor receptor α (PDGFRα) provides a therapeutic target independent of tumor cell PDGFRα expression in lung cancer xenografts. *Mol Cancer Ther* **11**, 2473-2482, doi:10.1158/1535-7163.mct-12-0431 (2012).
23 Primac, I. *et al.* Stromal integrin α11 regulates PDGFR-β signaling and promotes breast cancer progression. *The Journal of clinical investigation* **129**, 4609-4628, doi:10.1172/jci125890 (2019).
24 Peña, C. *et al.* STC1 expression by cancer-associated fibroblasts drives metastasis of colorectal cancer. *Cancer Res* **73**, 1287-1297, doi:10.1158/0008-5472.can-12-1875 (2013).
25 Laing, N. *et al.* Inhibition of platelet-derived growth factor receptor α by MEDI-575 reduces tumor growth and stromal fibroblast content in a model of non-small cell lung cancer. *Molecular pharmacology* **83**, 1247-1256, doi:10.1124/mol.112.084079 (2013).
26 Hong, L. *et al.* Expression of periostin in the serum of NSCLC and its function on proliferation and migration of human lung adenocarcinoma cell line (A549) in vitro. *Mol Biol Rep* **37**, 2285-2293, doi:10.1007/s11033-009-9721-1 (2010).
27 Li, M. *et al.* Periostin, a stroma-associated protein, correlates with tumor invasiveness and progression in nasopharyngeal carcinoma. *Clinical & experimental metastasis* **29**, 865-877, doi:10.1007/s10585-012-9465-5 (2012).
28 Okazaki, T. *et al.* Periostin is a negative prognostic factor and promotes cancer cell proliferation in non-small cell lung cancer. *Oncotarget* **9**, 31187-31199 (2018).
29 Underwood, T. J. *et al.* Cancer-associated fibroblasts predict poor outcome and promote periostin-dependent invasion in oesophageal adenocarcinoma. *The Journal of pathology* **235**, 466-477, doi:10.1002/path.4467 (2015).
30 González-González, L. & Alonso, J. Periostin: A Matricellular Protein With Multiple Functions in Cancer Development and Progression. *Front Oncol* **8**, 225, doi:10.3389/fonc.2018.00225 (2018).
31 Neri, S. *et al.* Podoplanin-expressing cancer-associated fibroblasts lead and enhance the local invasion of cancer cells in lung adenocarcinoma. *International Journal of Cancer* **137**, 784-796 (2015).
32 Nakamura, H. *et al.* Organoid culture containing cancer cells and stromal cells reveals that podoplanin-positive cancer-associated fibroblasts enhance proliferation of lung cancer cells. *Lung Cancer* **134**, 100-107, doi:10.1016/j.lungcan.2019.04.007 (2019).
33 Sakai, T. *et al.* Link between tumor-promoting fibrous microenvironment and an immunosuppressive microenvironment in stage I lung adenocarcinoma. *Lung Cancer* **126**, 64-71 (2018).
34 Wicki, A. & Christofori, G. The potential role of podoplanin in tumour invasion. *Br J Cancer* **96**, 1-5, doi:10.1038/sj.bjc.6603518 (2007).
35 Hoshino, A. *et al.* Podoplanin-positive fibroblasts enhance lung adenocarcinoma tumor formation: podoplanin in fibroblast functions for tumor progression. *Cancer Res* **71**, 4769-4779, doi:10.1158/0008-5472.Can-10-3228 (2011).

36 Ito, S. *et al.* Tumor promoting effect of podoplanin-positive fibroblasts is mediated by enhanced RhoA activity. *Biochem Biophys Res Commun* **422**, 194-199, doi:10.1016/j.bbrc.2012.04.158 (2012).
37 Xing, P. *et al.* High p-Smad2 expression in stromal fibroblasts predicts poor survival in patients with clinical stage I to IIIA non-small cell lung cancer. *World Journal of Surgical Oncology* **12** (2014).
38 Koukourakis, M. I. *et al.* Enhanced expression of SPARC/osteonectin in the tumor-associated stroma of non-small cell lung cancer is correlated with markers of hypoxia/acidity and with poor prognosis of patients. *Cancer Research* **63**, 5376-5380 (2003).
39 Drev, D. *et al.* Impact of Fibroblast-Derived SPARC on Invasiveness of Colorectal Cancer Cells. *Cancers (Basel)* **11**, doi:10.3390/cancers11101421 (2019).
40 Parekh, K. *et al.* Tenascin-C, over expressed in lung cancer down regulates effector functions of tumor infiltrating lymphocytes. *Lung Cancer* **47**, 17-29, doi:10.1016/j.lungcan.2004.05.016 (2005).
41 Gocheva, V. *et al.* Quantitative proteomics identify Tenascin-C as a promoter of lung cancer progression and contributor to a signature prognostic of patient survival. *Proc Natl Acad Sci U S A* **114**, E5625-e5634, doi:10.1073/pnas.1707054114 (2017).
42 Chen, Y. *et al.* Transforming growth factor-beta1 and alpha-smooth muscle actin in stromal fibroblasts are associated with a poor prognosis in patients with clinical stage I-IIIA nonsmall cell lung cancer after curative resection. *Tumour Biology* **35**, 6707-6713 (2014).
43 Koukourakis, M. I. *et al.* Different patterns of stromal and cancer cell thymidine phosphorylase reactivity in non small-cell lung cancer: Impact on tumour neoangiogenesis and survival. *British Journal of Cancer* **77**, 1696-1703 (1998).
44 Dai, S. *et al.* Two Gln187 mutants of human soluble APRIL inhibit proliferation of lung carcinoma A549 cells. *Acta Biochim Pol* **56**, 703-710 (2009).
45 Saito, R. A. *et al.* Forkhead box F1 regulates tumor-promoting properties of cancer-associated fibroblasts in lung cancer. *Cancer Res* **70**, 2644-2654, doi:10.1158/0008-5472.can-09-3644 (2010).
